# Antiviral Activity of Marine Bacterium *Paraliobacillus zengyii* Against Enterovirus 71 In Vitro and In Vivo

**DOI:** 10.3390/ijms26083500

**Published:** 2025-04-08

**Authors:** Qianjin Fan, Haoyue Huangfu, Lan Chen, Mengqi Jiao, Beijie Li, Zhijie Cao, Hui Sun, Xuelian Luo, Jianguo Xu

**Affiliations:** 1 Institute of Public Health, School of Medicine, Nankai University, Tianjin 300071, China; fanqianjin95@126.com (Q.F.);; 2 National Key Laboratory of Intelligent Tracking and Forecasting for Infectious Diseases, National Institute for Communicable Disease Control and Prevention, Chinese Center for Disease Control and Prevention, Beijing 102206, China; 3 Center of Reverse Microbial Etiology, School of Public Health, Shanxi Medical University, Taiyuan 030001, China; 4 Research Unite for Unknown Microbe, Chinese Academy of Medical Sciences & Peking Union Medical College, Beijing 100730, China

**Keywords:** *Paraliobacillus zengyii*, enterovirus 71, mouse model, antiviral activity, IFN response

## Abstract

Enterovirus 71 (EV71) is the major causative agent of hand, foot, and mouth disease (HFMD), leading to a serious health threat to young children. Probiotics are effective at treating or preventing gastrointestinal infections, especially viral infections. Probiotics against EV71 are mainly traditional lactic acid-producing bacteria, and most of them have been proven to be effective only in vitro. Here, we report that the marine bacterium *Paraliobacillus zengyii* X-1125 (*P. zengyii*) has promising anti-EV71 activity. The antiviral effect of *P. zengyii* against EV71 was assessed in different cell lines, and the viral RNA levels and titers were obviously reduced after treatment with *P. zengyii*. Furthermore, we established an EV71-infected mouse model to evaluate its antiviral efficacy in vivo. The oral administration of *P. zengyii* significantly decreased the viral loads in the hindlimb muscles, spleens, and ileums. Further research revealed that *P. zengyii* enhances the expression of type I interferon (IFN-I) in EV71-infected cells. Similarly, transcriptome analysis indicated that the expression of interferon-stimulated genes (ISGs) in EV71-infected mice significantly increased after *P. zengyii* treatment. Taken together, the results of this study indicated that *P. zengyii* markedly reduces EV71 infection by regulating the IFN response both in vivo and in vitro, providing a potential means to work against EV71 infection.

## 1. Introduction

Enterovirus 71 (EV71) belongs to the genus Enterovirus of the family Picornaviridae and can cause hand, foot, and mouth disease (HFMD) in infants and children younger than five years of age [1]. EV71 has caused multiple outbreaks since it was first isolated from a sick child in California in 1969 and has spread around the world, particularly into China and Southeast Asia [2,3,4]. Evidence has demonstrated that EV71 spreads from person to person predominantly through the fecal–oral route, including direct contact and indirect contact via contaminated food, water, or environmental surfaces [5,6]. Notably, the initial colonization and replication of EV71 within the gastrointestinal tract represent pivotal steps in the establishment of infection. This highlights the potential of gastrointestinal-targeted intervention strategies to effectively reduce viral transmission.

Probiotics often colonize in the gastrointestinal tract and are considered safe options for treating gastrointestinal diseases and improving overall human health [7,8,9]. Research has shown that probiotics can exert probiotic effects by producing beneficial metabolites, protecting against pathogens, enhancing the epithelial barrier, and improving the immune system [10,11,12]. In terms of antivirals, probiotics act mainly by modulating inflammatory cytokines or inducing type I interferon (IFN-I) [13,14]. Numerous probiotics, including *Lactobacillus plantarum*, *Bifidobacterium bifidum*, *Lactobacillus reuteri*, and *Lactobacillus casei*, have been reported to have anti-EV71 activity in vitro [15,16]; these probiotics are primarily *Lactobacillus* spp. and *Bifidobacterium* spp. In addition, other genera may also play a role in resisting EV71 infection.

Marine probiotics isolated from marine-related environments are used to improve aquaculture and human health [17,18,19]. Recent studies have shown that marine bacteria exhibit significant antiviral potential [20,21]. The genus *Paraliobacillus* is a Gram-positive, rod-shaped, facultatively anaerobic, spore-forming, slightly halophilic, and extremely halotolerant bacterium isolated from decomposing marine algae [22]. *Paraliobacillus zengyii* X-1125 (*P. zengyii*), a new member of *Paraliobacillus*, was isolated from the Qinghai–Tibetan Plateau [23]. Previous studies have demonstrated that *P. zengyii* possesses notable antiviral effects against influenza A virus (IAV) [24]. In this study, we demonstrated that *P. zengyii* has potential antiviral effects against EV71 by inducing IFN-I expression in vitro. Meanwhile, we also developed a 14-day-old EV71-infected suckling mouse model to further confirm its antiviral effect in vivo. This study provides new insights into EV71 infection prevention by probiotic therapy.

## 2. Results

### 2.1. P. zengyii Inhibits EV71 Infection In Vitro

EV71 can infect many cell types, including intestinal cells, rhabdomyosarcoma cells, etc. [25,26]. Here, three different cell lines (HT-29, Caco-2, and RD) were selected to explore the potential antiviral activity of *P. zengyii*. First, we evaluated the effect of *P. zengyii* on the viability of the aforementioned cell lines. The results indicated that live bacteria with a MOI greater than 500 appeared to be toxic to the three different cell lines (Figure S1).

**Figure 1:**
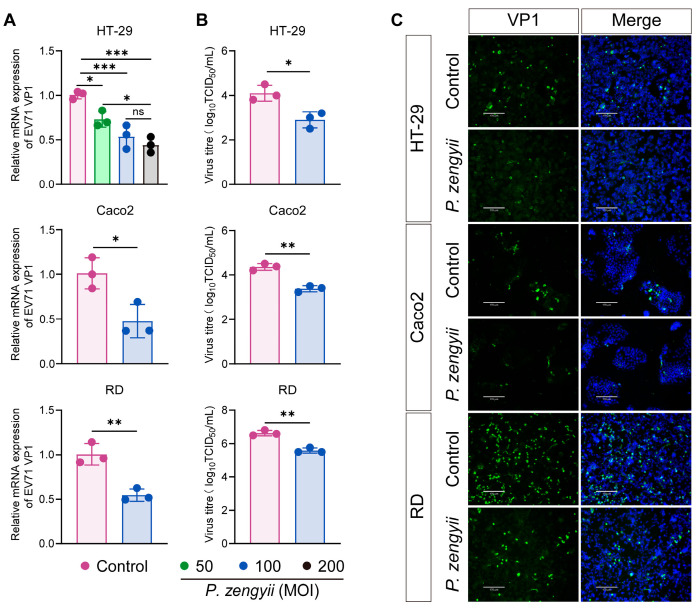
*P. zengyii* downregulates EV71 replication in multiple cell lines. (**A**) EV71 RNA levels relative to those of GAPDH were detected via RT–qPCR. (**B**) The virus titer was determined via a TCID_50_ assay. (**C**) Intracellular EV71 VP1 was examined by IFA. Multiple cell lines, including HT-29, Caco2, and RD cells, were used. All the cell lines were pretreated with or without *P. zengyii* for 24 h and then infected with EV71 for 24 h. The nuclei were labeled with DAPI, and images were taken using fluorescence microscopy (ECHO Revolve). The scale bars represent 170 μm. The data are shown as the means ± SDs of three independent experiments. One-way ANOVA and two-tailed unpaired Student’s *t* tests were used. ns: not significant; * *p* < 0.05, ** *p* < 0.01, *** *p* < 0.001.

Then, we examined the antiviral activity of *P. zengyii* during pretreatment. We investigated the potential inhibitory effects of different concentrations of *P. zengyii* in HT-29 cells. As shown in Figure 1A, the antiviral effect of *P. zengyii*, at an MOI of 200 was slightly higher than at an MOI of 50, but the difference from an MOI of 100 was not particularly obvious, according to RT-qPCR results. Therefore, subsequent in vitro assays were conducted with *P. zengyii* at an MOI of 100.

We next investigated the antiviral activity of *P. zengyii* in other cell lines. The results indicated that EV71 replication was inhibited by *P. zengyii*, and the inhibition rate was nearly 50%, as determined via RT–qPCR (Figure 1A). A tissue culture infectious dose 50% (TCID_50_) assay revealed that the virus titer was 1.1 ± 0.1 log10 TCID_50_/mL lower than that of the control (Figure 1B). To further validate the antiviral activity of *P. zengyii* against EV71, an immunofluorescence assay was performed. As shown in Figure 1C, the VP1 protein of EV71 was marked with green fluorescence, and the fluorescence signal was significantly reduced after pretreatment with *P. zengyii*. In addition, we also observed that the heat-inactivation of *P. zengyii* (MOI = 100) at 100 °C for 30 min resulted in the loss of its protective effect against EV71 infection (Figure S2A,B). These data indicate that live *P. zengyii* displays significant antiviral activity against EV71 in vitro.

### 2.2. P. zengyii Induces IFN-I Expression

**Figure 2:**
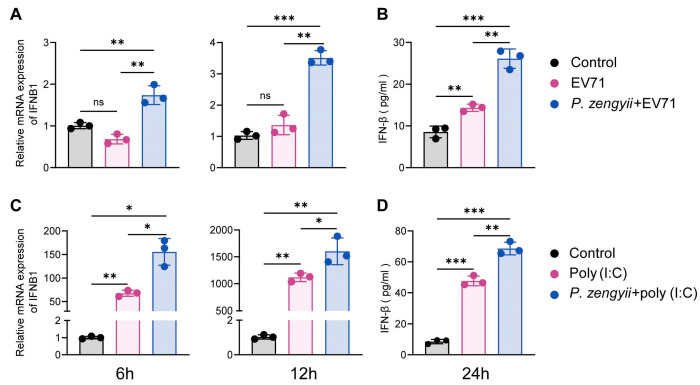
*P. zengyii* enhances the production of IFN-β in HT-29 cells. (**A**,**C**) IFN-β mRNA levels were measured via RT–qPCR and normalized to GAPDH levels after 6 and 12 h of treatment. (**B**,**D**) IFN-β protein levels were measured by ELISA after 24 h of treatment. HT-29 cells were pretreated with or without *P. zengyii* (MOI of 100) for 24 h and then infected with EV71 or transfected with poly(I:C) (0.5 μg/mL) for 6, 12, or 24 h. The control represents untreated HT-29 cells. The data are shown as the means ± SDs of three independent experiments. Two-tailed unpaired Student’s *t* tests were used. ns: not significant; * *p* < 0.05, ** *p* < 0.01, *** *p* < 0.001.

IFN-I potently inhibits virus replication [27]. We hypothesized that *P. zengyii* protects against EV71 infection by promoting the production of IFN-I. We first examined the expression of IFNB1 mRNA after the treatment of *P. zengyii* alone in HT-29 cells, and found that *P. zengyii* increased the expression of IFNB1 to a certain extent, but there was no statistical difference (Figure S3). Next, we detected the levels of IFN-β in HT-29 cells infected with EV71 after *P. zengyii* pretreatment. Surprisingly, the expression of IFNB1 mRNA was significantly upregulated at 6 h post-infection (hpi) and 12 hpi following preincubation with *P. zengyii* (Figure 2A). Moreover, IFN-β protein levels were greater in the treated group than in the untreated group (Figure 2B). Poly(I:C), a synthetic double-stranded RNA (dsRNA), has been widely used to mimic viral infection [28]. We examined the effect of *P. zengyii* pretreatment on the expression of IFN-β in response to poly(I:C) stimulation. Consistent with the results of viral infection, *P. zengyii* further promoted poly(I:C)-induced IFN-β production (Figure 2C,D). Taken together, these findings show that *P. zengyii* contributes to the production of IFN-I after stimulation with dsRNA or infection with EV71.

### 2.3. Establishment of EV71-Infected Mouse Model

**Figure 3:**
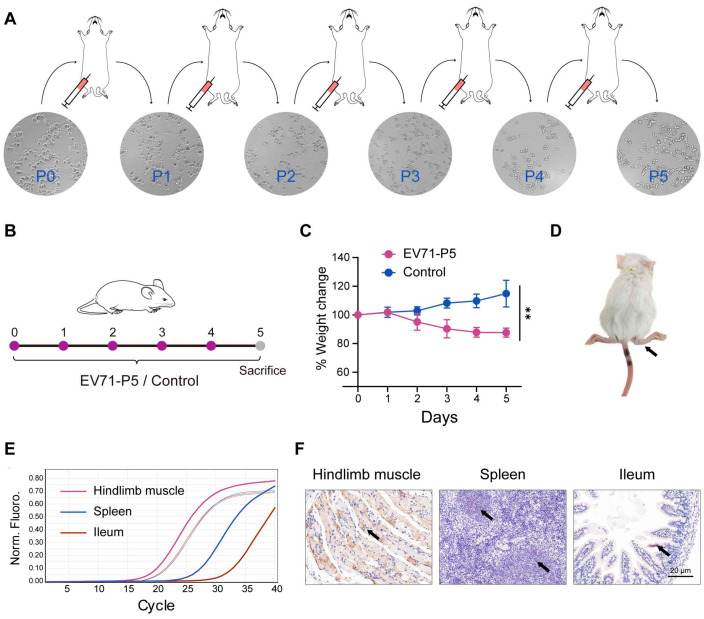
The establishment of the EV71 infection mouse model. (**A**) A schematic diagram of EV71 serial passaging from passages zero to five (P0-P5). Magnification on the images is 10×. (**B**) A schematic description of the intraperitoneal injection of EV71-P5 or DMEM into 14-day-old BALB/c mice (n = 5). (**C**) Changes in the body weights of the mice were recorded for 5 days after EV71-P5 infection, and the changes in body weight over time are expressed as a percentage of the original weight. (**D**) EV71-P5-infected mice presented hindlimb paralysis (black arrow). (**E**) The viral genomes detected by qPCR in mice hindlimb muscles, spleens, and ileums. The thin line indicates the internal reference gene GAPDH, and the thick line indicates EV71 VP1. (**F**) IHC analysis indicated that the viral antigen was diffusely distributed in the hindlimb muscles, spleens, and ileums (black arrow). The scale bars indicated 20 μm. Two-tailed unpaired Student’s *t* tests were used. ** *p* < 0.01.

We further explored the antiviral effects of *P. zengyii* in vivo. First, we set out to establish a mouse model of EV71 infection using 9- and 14-day-old suckling mice. EV71-P0 was intraperitoneally injected into 9-day-old suckling mice to obtain EV71-P1, and the EV71-P5 strain was obtained through the alternate passaging of the virus in RD cells and 14-day-old suckling mice (Figure 3A). Viral titers of P0-P5 were quantified and are summarized in Table 1. Furthermore, we infected 14-day-old BALB/c mice with the EV71-P5 strain (Figure 3B). The mice presented obvious disease symptoms at 5 days post-EV71-P5 infection, including weight loss, messy hair, and hindlimb paralysis (Figure 3C,D). We next detected viral RNA in multiple tissues. The viral RNA of the hindlimb muscles, spleens, and ileums was detected via qPCR (Figure 3E). Strikingly, much higher viral RNA levels were detected in the hindlimb muscles than in the other two tissues, and immunohistochemistry (IHC) staining for viral antigens confirmed this finding (Figure 3F). In addition, one mouse died 4 days after infection and one died after 5 days. During the 5 days of clinical observation, the control group showed no clinical signs, and no viral RNA was detected. These results suggest that we successfully established an EV71-infected mouse model of infection, that was confirmed in the hindlimb muscles, spleens, and ileums of approximately 14-day-old BALB/c suckling mice.

### 2.4. P. zengyii Exhibits Antiviral Activity in EV71-Infected Mice

**Figure 4:**
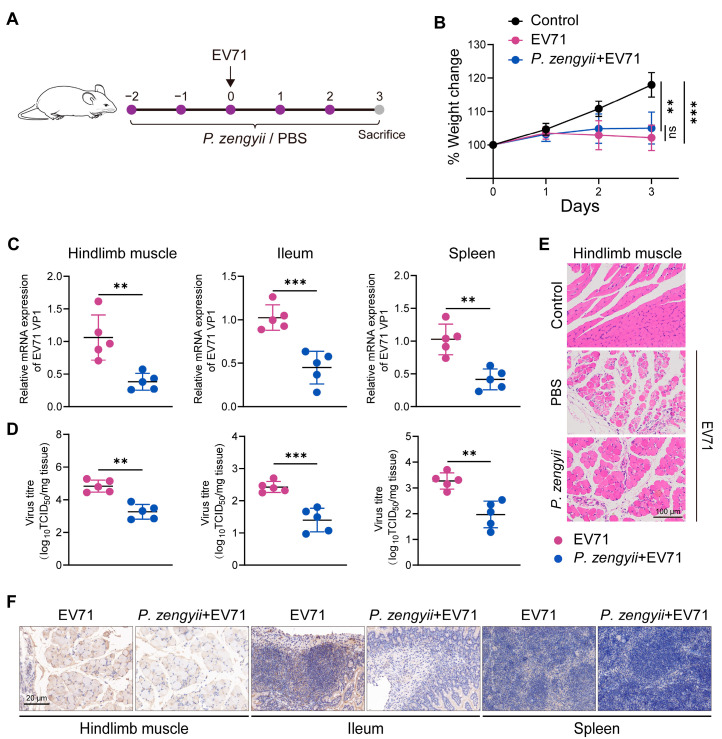
*P. zengyii* reduces EV71 infection in suckling mice. (**A**) A schematic representation of the animal experimental setup. Fourteen-day-old suckling mice were infected with 10^8^ TCID_50_ of EV71 and treated with *P. zengyii* or PBS from 2 days before infection until 3 days after infection. (**B**) Body weight was measured daily after EV71 infection and normalized to the corresponding initial weight. (**C**) The viral RNA levels in the hindlimb muscle, ileum, and spleen tissues of the infected mice were analyzed by RT-qPCR. (**D**) The viral titers of the hindlimb muscle, ileum, and spleen tissues of the infected mice were assessed via a TCID_50_ assay. (**E**) The HE staining of the hindlimb muscles. The scale bars indicate 100 μm. (**F**) The detection of the EV71 VP1 antigen in the mice hindlimb muscles, ileums, and spleens using IHC. The scale bars indicate 20 μm. GAPDH was used as an internal control for RT–qPCR. Two-tailed unpaired Student’s *t* tests were used. ns: not significant; ** *p* < 0.01, *** *p* < 0.001.

We next examined the antiviral activity of *P. zengyii* in a 14-day-old suckling mouse model (Figure 4A). In contrast to the uninfected controls, which did not exhibit weight loss, EV71-infected mice lost weight starting on Day 2 post-infection. However, the body weight loss was alleviated in EV71-infected mice orally administered *P. zengyii* compared with the untreated group (Figure 4B). Although not statistically significant, there was a clear trend. Additionally, we detected the impact of *P. zengyii* on viral RNA levels and virus titers within mouse tissues. Compared with the EV71 group, *P. zengyii* treatment significantly diminished EV71 loads in the hindlimb muscles, spleens, and ileums (Figure 4C). The virus titers of the above tissues also decreased in the *P. zengyii* treatment group (Figure 4D). Moreover, the hindlimb muscles were histologically examined via Hematoxylin and Eosin (HE) staining. The hindlimb muscles of the EV71-infected mice presented significant atrophy, dissolution, and increased infiltration of inflammatory cells, whereas those of the *P. zengyii*-treated mice presented alleviation of pathological damage (Figure 4E). In addition, we detected the EV71 viral antigen in hindlimb muscle, spleen, and ileum tissues via IHC. The amount of antigen was reduced significantly in the mice treated with *P. zengyii* (Figure 4F). No mice died during the experiment, and no viral antigen was detected in the uninfected control group. Altogether, these results underscore that *P. zengyii* confers protective effects against EV71 infection in suckling mice.

### 2.5. P. zengyii Enhances ISG Production in Spleens of EV71-Infected Mice

**Figure 5:**
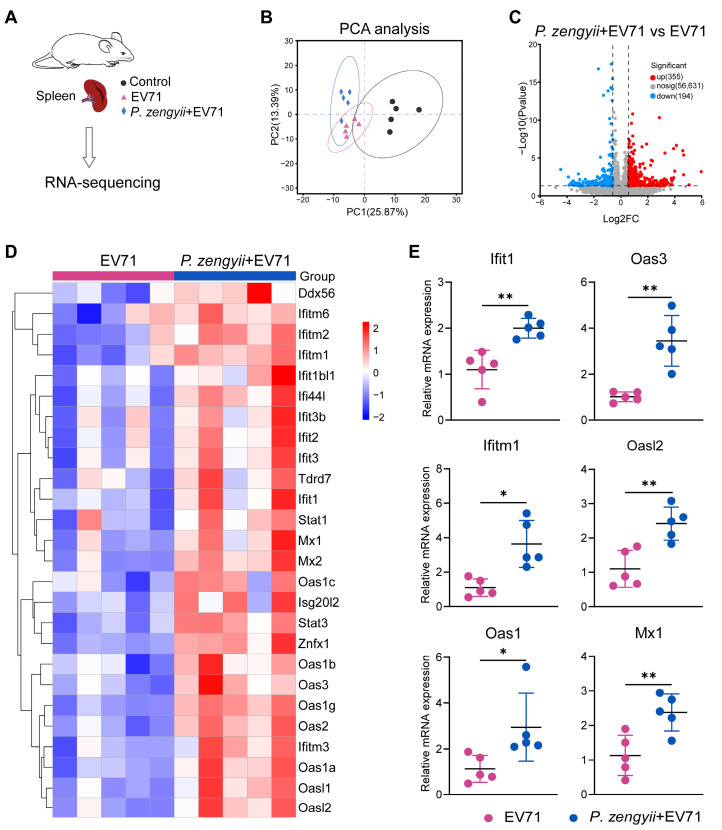
*P. zengyii* upregulates ISG expression in EV71-infected suckling mice. (**A**) A schematic diagram showing the spleen transcriptome analysis. (**B**) The PCA plot of the spleen transcriptome from the RNA sequencing analysis. (**C**) A volcano plot showing the upregulated (red), downregulated (blue) genes, and nonsignificant (gray) genes in the spleen tissues of EV71-infected mice treated with *P. zengyii* compared with those of the untreated mice. Fold change (FC) > 1.5; *p* value < 0.05. (**D**) A heatmap of some expressed ISGs. (**E**) The RT–qPCR expression of Ifit1, Oas3, Ifitm1, Oasl2, Oas2, and Mx1 in the spleens of EV71-infected mice treated with or without *P. zengyii*. GAPDH was used as an internal control for RT–qPCR. Two-tailed unpaired Student’s *t* tests were used. * *p* < 0.05, ** *p* < 0.01.

To further analyze the complete expression profile of *P. zengyii* treatment in EV71-infected mice, we measured the transcriptomes of mouse spleens (Figure 5A). Principal component analysis (PCA) revealed pronounced transcriptomic changes in the three groups (Figure 5B). Further, compared with the EV71 group, the volcano plot revealed 355 upregulated genes and 194 downregulated genes in the P. zengyii + EV71 group (Figure 5C). KEGG enrichment analysis revealed that the differentially expressed genes (DEGs) were enriched mainly in signal transduction (37 genes), signaling molecules and interactions (29 genes), the immune system (25 genes), cancer (23 genes), and viral infectious diseases (21 genes) (Figure S4). IFN suppresses viral infection by inducing the expression of interferon-stimulated genes (ISGs) [29]. We observed the strong upregulation of multiple ISGs, such as Ddx56, the IFITM family, the IFIT family, Mx1, Mx2, the OAS family, Stat1, Stat3, Isg20l2, Tdrd7, and Znfx1 (Figure 5D). We then verified the expression levels of some ISGs (Ifit1, Oas3, Ifitm1, Oasl2, Oas2, and Mx1) by RT–qPCR (Figure 5E). The above results show that the EV71-infected mice administered *P. zengyii* presented increased ISG expression, which reduced EV71 infection.

## 3. Discussion

In our study, we investigated the antiviral effects of *P. zengyii*, which targets EV71. *P. zengyii* is different from common lactic acid bacteria and is a member of a marine bacterial genus isolated from extreme environments. Compared to conventional probiotics, such as the reported antiviral effects of *Lactobacillus reuteri* against EV71, this study demonstrates that *P. zengyii* exhibits a more pronounced dose advantage. *Lactobacillus reuteri* requires a bacterial suspension concentration of 10^10^ CFU to exhibit mild antiviral activity [16]. In contrast, our study found that *P. zengyii* at a concentration of 3 × 10^7^ CFU (MOI = 100) already showed significant inhibition. This may be due to *P. zengyii* being isolated from extreme environments, which often exhibit unique biological characteristics, such as enhanced survival rate, better stability, and the production of distinctive functional metabolites [30,31,32]. Bacteria in similar environments have been reported to have antibiofilm [33], antioxidant [34], and antibacterial [35] effects, and have been used to treat cancer and to improve gastrointestinal disorders [19]. This study is the first to report that *P. zengyii*, as a bacterium from extreme environments, may have potential immunomodulatory effects and antiviral activity. These findings were confirmed by establishing an EV71-infected mouse model and the study of in vitro cells, enriching the sources of probiotics with antiviral effects and exploring the probiotic potential of this marine bacterial genus.

EV71 is one of the main causative pathogens of HFMD [36]. Many studies have initially demonstrated the anti-EV71 effects of probiotics, but few studies have been conducted in vivo. One reason for this is the lack of suitable animal models. Nonhuman primates can adequately develop symptoms of EV71 infection, but they are difficult to use because of ethical concerns and high maintenance costs [37,38,39]. Mouse models that are too young are not suitable for the oral administration of probiotics [40]. Moreover, considering that probiotics exert their antiviral effects mainly through regulating the host immune response, immunodeficient mouse models, transgenic mouse models, and hybrid mouse models are not suitable either [41,42,43]. We therefore chose a mouse model dependent on a mouse-adapted EV71 strain to evaluate the in vivo antiviral efficacy of *P. zengyii*.

We attempted to establish a mouse model of EV71 infection in approximately 14-day-old BALB/c mice by reusing hindlimb muscles for serial intraperitoneal injection–adaptation cycles. The mice displayed hindlimb paralysis on Day 4 after EV71-P5 strain infection, and the virus titers in the hindlimb muscles were very high, which is similar to the clinical symptoms of acute flaccid paralysis and is consistent with previous results [44,45]. However, we failed to detect EV71 RNA in the brain tissue, possibly because the mouse-adapted EV71 strain was generated from the hindlimb muscle tissue of the mice rather than the brain tissue [46]. Surprisingly, viral RNA and antigens were detected in the mice spleens and ileums. The spleen is the largest immune organ in humans and animals and might reflect the host immune status [47,48]. In light of these data, this infection model was suitable for studying the anti-EV71 ability of probiotics in vivo.

Previous studies have indicated that IFN-β plays an important role in multiple cells against EV71 infection [49,50]. Here, we found that *P. zengyii* significantly reduced EV71 infection in different cell lines and that *P. zengyii* increased IFN-β levels in EV71-infected HT-29 cells. These findings suggest that *P. zengyii* may exert antiviral effects by promoting the production of IFN-β. Interestingly, treatment with *P. zengyii* also promoted poly(I:C)-induced IFN-β production, indicating that *P. zengyii* may have broad-spectrum antiviral effects. The results of our study are similar to those of previous studies [51,52,53]. We further investigated the antiviral effects of *P. zengyii* against EV71 in mice. Compared with no treatment, treatment with *P. zengyii* reduced the viral titers in hindlimb muscle, spleen, and ileum tissues. More interestingly, the transcriptome results revealed numerous ISGs induced by *P. zengyii* in the spleens of EV71-infected mice. These results are mostly in agreement with the in vitro results, indicating that *P. zengyii* can reduce EV71 infection through modulating IFN response. Importantly, some ISGs (such as OAS) have been shown to inhibit EV71 infection [49]. Whether other ISGs directly suppress EV71 infection requires further study. It is noteworthy that we further observed that *P. zengyii* can directly interact with the EV71 virus, thereby inhibiting its activity. Additionally, we found that heat-inactivation of *P. zengyii* led to the loss of its antiviral effect, indicating that the antiviral activity may depend on specific metabolites produced in its active state. Further investigation is required to elucidate these underlying mechanisms. There are several potential limitations that exist in this study. Given that probiotics exert their beneficial effects via several mechanisms [54,55,56], more and more detailed mechanisms still need to be further explored in the future. While our current study utilized intraperitoneal viral challenge to evaluate antiviral efficacy, this approach does not fully recapitulate the natural oral–fecal transmission route of human EV71 infections. Thus, future research will focus on utilizing humanized SCARB2 transgenic mice to further evaluate the antiviral effects of *P. zengyii* in an oral infection model.

## 4. Materials and Methods

### 4.1. Cells, Viruses, and Bacteria

HT-29, Caco2, and RD cells were cultured in DMEM (Gibco, CA, USA) supplemented with 10% fetal bovine serum (FBS) (Vivacell, Shanghai, China) at 37 °C in a humidified atmosphere with 5% CO_2_. The EV71 strain was kindly gifted to us by Professor Wenkuan Liu (State Key Laboratory of Respiratory Diseases, National Clinical Research Center for Respiratory Disease, The First Affiliated Hospital of Guangzhou Medical University, Guangzhou Institute of Respiratory Health, Guangzhou, China). Viral titers in culture supernatants were analyzed by a TCID_50_ method in RD cells. *P. zengyii* was isolated and stored in our laboratory [23]. The bacterial strain was usually grown in BHI media supplemented with 3% (*w*/*v*) NaCl at an optimal temperature of 28 °C.

### 4.2. Cell Viability Assay

To determine the possible cytotoxic effects of *P. zengyii*, live bacteria (MOIs of 10, 100, 500, 1000, 2000, and 5000) were added to HT-29, Caco2, and RD cells (3 × 10^4^ cells/well) in 96-well plates for 24 h. Cell viability was determined with a Cell Counting Kit-8 (CCK-8) (Dojindo, Tokyo, Japan) assay according to the manufacturer’s instructions. The relative percentage (%) of cell survival with respect to that of the control wells containing untreated cells was calculated. The values were corrected for background fluorescence obtained with media only.

### 4.3. Antiviral Activity Assays

We aimed to study the modulation of host immune response by *P. zengyii*. In order to exclude the direct impact of *P. zengyii* on EV71, we set up a pretreatment experiment of *P. zengyii*. HT-29, Caco2, and RD cells (3 × 10^5^ cells/well) were seeded onto 12-well plates and pretreated with *P. zengyii* for 24 h. Afterwards, the cells were washed three times with PBS to remove bacteria and infected with EV71 (MOI = 0.01) for 1 h. Then, the cells were washed and incubated with maintenance medium containing 2% FBS, 100 U/mL penicillin, and 100 µg/mL streptomycin at 37 °C for the indicated time periods.

### 4.4. Mouse Infection Model

BALB/c suckling mice were purchased from Vital River Laboratory Animal Technology Co., Ltd. (Beijing, China), and allowed to adapt to the new laboratory conditions for 3 days. The following procedure was performed according to previous studies, with some modifications [40,44]. Nine-day-old BALB/c mice were injected with 100 µL of EV71 (P0) intraperitoneally. After 3 days, the mice were euthanized, and the hindlimb muscles were harvested. Then, the samples were ground to obtain tissue grinding fluid, which was subsequently diluted and inoculated into RD cells. When more than 90% of the cells showed cytopathic effects (CPEs), the virus was harvested and named EV71-P1, and the viral titer was measured. The EV71-P1 strain was subsequently injected intraperitoneally into 14-day-old BALB/c mice, and the above operation was subsequently repeated until the EV71-P5 strain was obtained. The challenge doses for EV71 P0-P4 were all 10^7^ TCID_50_ per mouse. EV71-P5 strain (10^8^ TCID_50_/mouse, 100 μL) was then intraperitoneally injected into 14-day-old BALB/c mice, and body weight changes were recorded. The control group was instead given DMEM intraperitoneally, with 5 mice in each group. On Day 5, the mice were dissected, and the same amounts of the major organs (heart, lung, liver, spleen, kidney, brain, small intestine, large intestine and hindlimb muscle) were collected for viral RNA detection or immunohistochemistry (IHC) analysis.

### 4.5. Experimental Animals

Fourteen-day-old BALB/c suckling mice were used to evaluate whether *P. zengyii* protects mice from EV71 infection. The mice were randomly divided into three groups (n = 5 per group): the control, EV71, and *P. zengyii* + EV71 groups. The mice in the control and EV71 groups were orally administered PBS (100 μL) every day. Mice in the *P. zengyii* + EV71 group were orally administered *P. zengyii* (5 × 10^8^ CFU/mouse, 100 μL) daily. The mice in the EV71 and *P. zengyii* + EV71 groups were infected with EV71 (10^8^ TCID_50_/mouse, 100 μL) via intraperitoneal injection on Day 3 after intragastric administration (set as Day 0), and the control group was instead given DMEM intraperitoneally. After 3 days of EV71 infection, the mice were sacrificed, and their hindlimb muscles, spleens, and ileums were collected for subsequent analysis. HE staining was performed according to previous reports [52]. Body weights were recorded daily from the infection day (Day 0).

### 4.6. TCID_50_ Assay

The virus titers were determined via a TCID_50_ assay in the RD cells. Briefly, RD cells were seeded (3 × 10^4^ cells per well in 100 μL) in a 96-well flat-bottom plate for 24 h. The tissue homogenate was centrifuged, and the supernatants were collected. Then, tenfold serial dilutions of cell or homogenized supernatants were inoculated onto the cells. The virus-containing medium was removed after 1 h, and the culture medium was replaced with fresh medium to continue culturing. The plates were incubated at 37 °C for 5 days before the CPEs were measured under a light microscope. The TCID_50_ value was calculated by the Reed–Muench method.

### 4.7. RNA Extraction, RT–qPCR, and RNA Sequencing

After the mouse tissues were isolated, cold PBS was added for grinding, and the tissue homogenate was collected. TRIzol reagent (Invitrogen, Cartsbad, CA, USA) was used to isolate the total RNA from the homogenates or cells. A total of 0.5 μg of RNA was reverse-transcribed to cDNA with a PrimeScript RT Reagent Kit (TaKaRa, Kyoto, Japan), and quantitative real-time PCR was then performed with SYBR Green qPCR Master Mix (TaKaRa, Kyoto, Japan). The primers used are listed in Table 2. The mRNA levels of the target genes were normalized to the GAPDH level, and the relative mRNA levels were compared via the 2^−ΔΔCt^ method. RNA sequencing was performed by Majorbio (Shanghai, China), and the data analysis was performed on the Majorbio cloud platform.

### 4.8. Immunofluorescence and Immunohistochemistry Analyses

After EV71 infection, HT-29, Caco2, and RD cells were fixed with 4% paraformaldehyde for 30 min and then permeabilized with 0.22% Triton X-100. Next, the cells were blocked with 5% skimmed milk. After being washed with PBS, the HT-29, Caco2, and RD cells were incubated with a rabbit anti-enterovirus 71 VP1 polyclonal antibody (GTX132339) (GeneTex, Irvine, CA, USA) for 1 h, followed by incubation with a goat anti-rabbit FITC secondary antibody (ZSGB-BIO, Beijing, China) in the dark for 1 h. DAPI (Lablead, Beijing, China) was then added for 5 min at room temperature. The images were observed under a fluorescence microscope (ECHO Revolve, San Diego, CA, USA). For the IHC studies, tissue samples from the above mouse studies were fixed, paraffin-embedded, sectioned at 4 μm, and deparaffinized. The sections were stained with an anti-enterovirus 71 VP1 polyclonal antibody and detected with a Histostain-Plus IHC HRP Kit and DAB (Thermo Fisher, Waltham, MA, USA).

### 4.9. Transfection of Poly(I:C) and Detection of IFN-β

HT-29 cells were seeded (3 × 10^5^ cells per well) in a 12-well plate. When the cells were grown to approximately 70–80% confluency, poly(I:C) (Invitrogen, Cartsbad, CA, USA) was transfected into the cells using Lipofectamine 2000 (Invitrogen, Cartsbad, CA, USA) according to the manufacturer’s protocol. The cells and culture supernatants were individually collected at the indicated time points. The concentrations of IFN-β in the culture supernatants were measured using a specific human IFN-β ELISA kit (Proteintech, Wuhan, China). The ELISA was performed according to the manufacturer’s recommendations, and the results are expressed as pg/mL.

### 4.10. Statistical Analysis

Experimental data from at least three independent experiments were presented as the means ± standard deviation (SD) and analyzed with GraphPad Prism 8.0. Differences between the different groups were determined using Student’s *t* test or one-way ANOVA. *p* values < 0.05 were considered statistically significant, and * indicates *p* < 0.05, ** indicates *p* < 0.01, and *** indicates *p* < 0.001.

## 5. Conclusions

In summary, our study indicated that the marine bacterium *P. zengyii* efficiently inhibited EV71 infection in HT-29, Caco-2, and RD cells, as well as in a suckling mouse model. Moreover, we confirmed that *P. zengyii* can reduce EV71 infection by regulating the IFN response. This is the first report of marine bacteria with anti-EV71 activity. Our results suggest that *P. zengyii* has the potential to be a new tool for preventing EV71 infection.

## Figures and Tables

**Table 1:** Virus titers of EV71 P0-P5.

Passage Numbers	P0	P1	P2	P3	P4	P5
Virus titers	1.61 × 10^8^ TCID_50_/mL	1.79 × 10^8^ TCID_50_/mL	6.40 × 10^8^ TCID_50_/mL	1.0 × 10^9^ TCID_50_/mL	1.28 × 10^9^ TCID_50_/mL	1.79 × 10^9^ TCID_50_/mL

**Table 2:** Primers used in this study.

Gene Name ^1^	Forward (5′-3′)	Reverse (5′-3′)
hGAPDH	GACACCCACTCCTCCACCTTT	TTGCTGTAGCCAAATTCGTTGT
hIFNB1	GCCGCATTGACCATCTAT	TAGACATTAGCCAGGAGGTT
EV71-VP1	AAGGTTCCAGCACTCCAAGC	TCTCCAACTAATCCCGCCC
mGAPDH	AGGTCGGTGTGAACGGATTTG	TGTAGACCATGTAGTTGAGGTCA
mIfItm1	GACAGCCACCACAATCAACAT	CCCAGGCAGCAGAAGTTCAT
mOasl2	TTGTGCGGAGGATCAGGTACT	TGATGGTGTCGCAGTCTTTGA
mMx1	GACCATAGGGGTCTTGACCAA	AGACTTGCTCTTTCTGAAAAGCC
mIfit1	CTGAGATGTCACTTCACATGGAA	GTGCATCCCCAATGGGTTCT
mOas1	GAAGAGGCTGATGTGTGGCT	TGTCCAGTTCTCTTCTACCTGC
mOas3	GAACAGCAAGGTGGCCTTTG	CCTCAGGAGTCCTTGTGCAG

^1^ m: mouse; h: human.

## Data Availability

The original contributions presented in the study are included in the article; further inquiries can be directed to the corresponding authors.

## Supplementary Materials

The following supporting information can be downloaded at: https://www.mdpi.com/article/10.3390/ijms26083500/s1.

## References

[1] Zhang H., Song Z., Zou J., Feng Y., Zhang J., Ren L., Zhang X., Hu Y., Yuan Z., Yi Z. (2020). An infectious clone of enterovirus 71(EV71) that is capable of infecting neonatal immune competent mice without adaptive mutations. Emerg. Microbes Infect..

[2] Wu C., Zhu G., Qiu F., Ren F., Lin B., Zhang D., Yang Q., Huang C. (2023). PLX8394, a RAF inhibitor, inhibits enterovirus 71 replication by blocking RAF/MEK/ERK signaling. Virol. Sin..

[3] Yip C.C., Lau S.K., Woo P.C., Yuen K.Y. (2013). Human enterovirus 71 epidemics: What’s next?. Emerg. Health Threat. J..

[4] Li Q., Zheng Z., Liu Y., Zhang Z., Liu Q., Meng J., Ke X., Hu Q., Wang H. (2016). 2C Proteins of Enteroviruses Suppress IKKβ Phosphorylation by Recruiting Protein Phosphatase 1. J. Virol..

[5] Solomon T., Lewthwaite P., Perera D., Cardosa M.J., McMinn P., Ooi M.H. (2010). Virology, epidemiology, pathogenesis, and control of enterovirus 71. Lancet Infect. Dis..

[6] Phyu W.K., Ong K.C., Wong K.T. (2017). Modelling person-to-person transmission in an Enterovirus A71 orally infected hamster model of hand-foot-and-mouth disease and encephalomyelitis. Emerg. Microbes Infect..

[7] Pant N., Marcotte H., Brüssow H., Svensson L., Hammarström L. (2007). Effective prophylaxis against rotavirus diarrhea using a combination of Lactobacillus rhamnosus GG and antibodies. BMC Microbiol..

[8] Toscano M., De Grandi R., Pastorelli L., Vecchi M., Drago L. (2017). A consumer’s guide for probiotics: 10 golden rules for a correct use. Dig. Liver Dis..

[9] Pan H., Guo R., Ju Y., Wang Q., Zhu J., Xie Y., Zheng Y., Li T., Liu Z., Lu L. (2019). A single bacterium restores the microbiome dysbiosis to protect bones from destruction in a rat model of rheumatoid arthritis. Microbiome.

[10] Ren Z., Peng L., Chen S., Pu Y., Lv H., Wei H., Wan C. (2021). Lactiplantibacillus plantarum 1201 Inhibits Intestinal Infection of *Salmonella enterica* subsp. enterica Serovar Typhimurium Strain ATCC 13311 in Mice with High-Fat Diet. Foods.

[11] Lee H., Jung K.B., Kwon O., Son Y.S., Choi E., Yu W.D., Son N., Jeon J.H., Jo H., Yang H. (2022). Limosilactobacillus reuteri DS0384 promotes intestinal epithelial maturation via the postbiotic effect in human intestinal organoids and infant mice. Gut Microbes.

[12] Zhou C., Fang X., Xu J., Gao J., Zhang L., Zhao J., Meng Y., Zhou W., Han X., Bai Y. (2020). Bifidobacterium longum alleviates irritable bowel syndrome-related visceral hypersensitivity and microbiota dysbiosis via Paneth cell regulation. Gut Microbes.

[13] Lu S., He S., Yue K., Mi J., Huang Y., Song L., Yang T., Ren Z., Ren L., Xu J. (2024). Lactobacillus plantarum GUANKE modulate anti-viral function of dendritic cells in mice. Int. Immunopharmacol..

[14] Kumova O.K., Fike A.J., Thayer J.L., Nguyen L.T., Mell J.C., Pascasio J., Stairiker C., Leon L.G., Katsikis P.D., Carey A.J. (2019). Lung transcriptional unresponsiveness and loss of early influenza virus control in infected neonates is prevented by intranasal Lactobacillus rhamnosus GG. PLoS Pathog..

[15] Choi H.-J., Song J.-H., Park K.-S., Baek S.-H., Lee E.-S., Kwon D.-H. (2010). Antiviral activity of yogurt against enterovirus 71 in vero cells. Food Sci. Biotechnol..

[16] Ang L.Y., Too H.K., Tan E.L., Chow T.K., Shek L.P., Tham E.H., Alonso S. (2016). Antiviral activity of Lactobacillus reuteri Protectis against Coxsackievirus A and Enterovirus 71 infection in human skeletal muscle and colon cell lines. Virol. J..

[17] Ravi A.V., Musthafa K.S., Jegathammbal G., Kathiresan K., Pandian S.K. (2007). Screening and evaluation of probiotics as a biocontrol agent against pathogenic Vibrios in marine aquaculture. Lett Appl. Microbiol..

[18] Madison D., Schubiger C., Lunda S., Mueller R.S., Langdon C. (2022). A marine probiotic treatment against the bacterial pathogen *Vibrio coralliilyticus* to improve the performance of Pacific (*Crassostrea gigas*) and Kumamoto (*C. sikamea*) oyster larvae. Aquaculture.

[19] Eze O.C., Berebon D.P., Emencheta S.C., Evurani S.A., Okorie C.N., Balcão V.M., Vila M.M.D.C. (2023). Therapeutic Potential of Marine Probiotics: A Survey on the Anticancer and Antibacterial Effects of *Pseudoalteromonas* spp.. Pharmaceuticals.

[20] Kim H.J., Park J.G., Moon K.S., Jung S.B., Kwon Y.M., Kang N.S., Kim J.H., Nam S.J., Choi G., Baek Y.B. (2024). Identification and characterization of a marine bacterium extract from *Mameliella* sp. M20D2D8 with antiviral effects against influenza A and B viruses. Arch. Virol..

[21] Lin S.C., Lehman C.W., Stewart A.K., Panny L., Bracci N., Wright J.L.C., Paige M., Strangman W.K., Kehn-Hall K. (2021). Homoseongomycin, a compound isolated from marine actinomycete bacteria K3-1, is a potent inhibitor of encephalitic alphaviruses. Antivir. Res..

[22] Ishikawa M., Ishizaki S., Yamamoto Y., Yamasato K. (2002). *Paraliobacillus ryukyuensis* gen. nov., sp. nov., a new Gram-positive, slightly halophilic, extremely halotolerant, facultative anaerobe isolated from a decomposing marine alga. J. Gen. Appl. Microbiol..

[23] Wang X., Yang J., Lu S., Lai X.H., Jin D., Pu J., Niu L., Zhu W., Liang J., Huang Y. (2019). *Paraliobacillus zengyii* sp. nov., a slightly halophilic and extremely halotolerant bacterium isolated from Tibetan antelope faeces. Int. J. Syst. Evol. Microbiol..

[24] Fan Q.J., Li B.J., Lu S.M., Yue K., Luo X.L., Xu J.G. (2025). Study on the Anti⁃Influenza Virus of Marine Probiotic Paraliobacillus zengyii CGMCC1.16464. Bingduxuebao.

[25] Dong Y., Liu J., Lu N., Zhang C. (2021). Enterovirus 71 Antagonizes Antiviral Effects of Type III Interferon and Evades the Clearance of Intestinal Intraepithelial Lymphocytes. Front. Microbiol..

[26] Lee C.H., Huang P.N., Mwale P.F., Wang W.C., Leu S.J., Tseng S.N., Shih S.R., Chiang L.C., Mao Y.C., Tsai B.Y. (2022). The Bottlenecks of Preparing Virus Particles by Size Exclusion for Antibody Generation. Int. J. Mol. Sci..

[27] Takahashi T., Nakano Y., Onomoto K., Yoneyama M., Ui-Tei K. (2020). LGP2 virus sensor enhances apoptosis by upregulating apoptosis regulatory genes through TRBP-bound miRNAs during viral infection. Nucleic Acids Res..

[28] Novak T., Hall M.W., McDonald D.R., Newhams M.M., Mistry A.J., Panoskaltsis-Mortari A., Mourani P.M., Loftis L.L., Weiss S.L., Tarquinio K.M. (2020). RIG-I and TLR4 responses and adverse outcomes in pediatric influenza-related critical illness. J. Allergy Clin. Immunol..

[29] Jimenez-Guardeño J.M., Apolonia L., Betancor G., Malim M.H. (2019). Immunoproteasome activation enables human TRIM5α restriction of HIV-1. Nat. Microbiol..

[30] Mythrayee R., Gayathri K.V., Ranjan A., Rajput V.D., Chauhan A., Prazdnova E.V.e., Minkina T., Zargar S.M. (2024). Survival Strategies of Extremophiles: Physiology and Biochemistry. Extremophiles for Sustainable Agriculture and Soil Health Improvement.

[31] Hui M.L., Tan L.T., Letchumanan V., He Y.W., Fang C.M., Chan K.G., Law J.W., Lee L.H. (2021). The Extremophilic Actinobacteria: From Microbes to Medicine. Antibiotics.

[32] Neifar M., Maktouf S., Ghorbel R., Jaouani A., Cherif A. (2015). Extremophiles as source of novel bioactive compounds with industrial potential. Biotechnology of Bioactive Compounds: Sources and Applications.

[33] El Aichar F., Muras A., Parga A., Otero A., Nateche F. (2022). Quorum quenching and anti-biofilm activities of halotolerant Bacillus strains isolated in different environments in Algeria. J. Appl. Microbiol..

[34] Yang T., Fan X., Li D., Zhao T., Wu D., Liu Z., Long D., Li B., Huang X. (2023). High Antioxidant Capacity of *Lacticaseibacillus paracasei* TDM-2 and *Pediococcus pentosaceus* TCM-3 from Qinghai Tibetan Plateau and Their Function towards Gut Modulation. Foods.

[35] Ran X., Li X., Xie X., Lei J., Yang F., Chen D. (2024). Effects of Probiotic Enterococcus faecium from Yak on the Intestinal Microflora and Metabolomics of Mice with Salmonella Infection. Probiotics Antimicrob. Proteins.

[36] Kobayashi K., Koike S. (2020). Cellular receptors for enterovirus A71. J. Biomed. Sci..

[37] Arita M., Shimizu H., Nagata N., Ami Y., Suzaki Y., Sata T., Iwasaki T., Miyamura T. (2005). Temperature-sensitive mutants of enterovirus 71 show attenuation in cynomolgus monkeys. J. Gen. Virol..

[38] Arita M., Nagata N., Iwata N., Ami Y., Suzaki Y., Mizuta K., Iwasaki T., Sata T., Wakita T., Shimizu H. (2007). An attenuated strain of enterovirus 71 belonging to genotype a showed a broad spectrum of antigenicity with attenuated neurovirulence in cynomolgus monkeys. J. Virol..

[39] Hashimoto I., Hagiwara A. (1982). Pathogenicity of a poliomyelitis-like disease in monkeys infected orally with enterovirus 71: A model for human infection. Neuropathol. Appl. Neurobiol..

[40] Wang H.Q., Jiang J.D., Li Y.H. (2013). Establishment of EV71 animal models with 2-week-old BALB/c mice. Yao Xue Xue Bao.

[41] Liao C.C., Liou A.T., Chang Y.S., Wu S.Y., Chang C.S., Lee C.K., Kung J.T., Tu P.H., Yu Y.Y., Lin C.Y. (2014). Immunodeficient mouse models with different disease profiles by in vivo infection with the same clinical isolate of enterovirus 71. J. Virol..

[42] Fujii K., Nagata N., Sato Y., Ong K.C., Wong K.T., Yamayoshi S., Shimanuki M., Shitara H., Taya C., Koike S. (2013). Transgenic mouse model for the study of enterovirus 71 neuropathogenesis. Proc. Natl. Acad. Sci USA.

[43] Liou A.T., Wu S.Y., Liao C.C., Chang Y.S., Chang C.S., Shih C. (2016). A new animal model containing human SCARB2 and lacking stat-1 is highly susceptible to EV71. Sci. Rep..

[44] Ong K.C., Devi S., Cardosa M.J., Wong K.T. (2010). Formaldehyde-inactivated whole-virus vaccine protects a murine model of enterovirus 71 encephalomyelitis against disease. J. Virol..

[45] Good C., Wells A.I., Coyne C.B. (2019). Type III interferon signaling restricts enterovirus 71 infection of goblet cells. Sci. Adv..

[46] Wang Y.F., Chou C.T., Lei H.Y., Liu C.C., Wang S.M., Yan J.J., Su I.J., Wang J.R., Yeh T.M., Chen S.H. (2004). A mouse-adapted enterovirus 71 strain causes neurological disease in mice after oral infection. J. Virol..

[47] He R., Zang J., Zhao Y., Liu Y., Ruan S., Zheng X., Chong G., Xu D., Yang Y., Yang Y. (2021). Nanofactory for metabolic and chemodynamic therapy: Pro-tumor lactate trapping and anti-tumor ROS transition. J. Nanobiotechnol..

[48] Yong T., Chen S., Xie Y., Shuai O., Li X., Chen D., Su J., Jiao C., Liang Y. (2018). Hypouricemic Effects of Extracts From Agrocybe aegerita on Hyperuricemia Mice and Virtual Prediction of Bioactives by Molecular Docking. Front. Pharmacol..

[49] Zheng B., Zhou X., Tian L., Wang J., Zhang W. (2022). IFN-β1b induces OAS3 to inhibit EV71 via IFN-β1b/JAK/STAT1 pathway. Virol. Sin..

[50] Huang H.I., Lin J.Y., Chen S.H. (2019). EV71 Infection Induces IFNβ Expression in Neural Cells. Viruses.

[51] Macpherson C., Audy J., Mathieu O., Tompkins T.A. (2014). Multistrain probiotic modulation of intestinal epithelial cells’ immune response to a double-stranded RNA ligand, poly(i·c). Appl. Environ. Microbiol..

[52] Kang N., Gao H., He L., Liu Y., Fan H., Xu Q., Yang S. (2021). Ginsenoside Rb1 is an immune-stimulatory agent with antiviral activity against enterovirus 71. J. Ethnopharmacol..

[53] Yu J., Dai Y., Fu Y., Wang K., Yang Y., Li M., Xu W., Wei L. (2021). Cathelicidin antimicrobial peptides suppress EV71 infection via regulating antiviral response and inhibiting viral binding. Antivir. Res..

[54] Li Q., Li L., Chen Y., Yu C., Azevedo P., Gong J., Yang C. (2022). Bacillus licheniformis PF9 improves barrier function and alleviates inflammatory responses against enterotoxigenic Escherichia coli F4 infection in the porcine intestinal epithelial cells. J. Anim. Sci. Biotechnol..

[55] Sang Y., Ren J., Aballay A. (2022). The transcription factor HLH-26 controls probiotic-mediated protection against intestinal infection through up-regulation of the Wnt/BAR-1 pathway. PLoS Biol..

[56] Deriu E., Liu J.Z., Pezeshki M., Edwards R.A., Ochoa R.J., Contreras H., Libby S.J., Fang F.C., Raffatellu M. (2013). Probiotic bacteria reduce salmonella typhimurium intestinal colonization by competing for iron. Cell Host Microbe.

