# Supplementary material for: Antiviral Activity of Marine Bacterium Paraliobacillus zengyii Against Enterovirus 71 In Vitro and In Vivo
Source: Int J Mol Sci. 2025 Apr 8;26(8):3500. doi: 10.3390/ijms26083500 (PMC12026459; doi:10.3390/ijms26083500)
Supplement: Supplementary file 1 [file ijms-26-03500-s001.zip › Supplementary Figure legends.docx]

**Supplementary Figure 1.** Effects of *P. zengyii* on cell viability. (A) HT-29 cells. (B) Caco2 cells. (C) RD cells. The cells were treated with different concentrations of *P. zengyii* (MOIs of 10–5000) at 37 °C for 24 h. Cell viability was measured via a CCK-8 assay. All the values were normalized to those of the control group, which represented 100% cell viability. The data are presented as the means ± SDs of three independent experiments.

**Supplementary Figure 2.** Heat inactivation of *P. zengyii* does not reduce EV71 infection in HT-29 cells. (A) EV71 RNA levels relative to those of GAPDH were detected via RT‒qPCR. (B) The virus titre was determined via a TCID50 assay. HT-29 cells were pretreated with or without heat-inactivated *P. zengyii* (MOI of 100) for 24 h and then infected with EV71 for 24 h. The data are shown as the means ± SDs of three independent experiments. Two-tailed unpaired Student’s t test was used. ns, not significant.

**Supplementary Figure 3.** Effects of the treatment of *P. zengyii* alone on IFN-β in HT-29 cells. (A, B) IFN-β mRNA levels were measured via RT‒qPCR and normalized to GAPDH levels after 12 and 24 h of *P. zengyii* treatment. Data are shown as the means ± SDs of three independent experiments. Two-tailed unpaired Student’s t test was used. ns, not significant.

**Supplementary Figure 4.** Histogram of the KEGG enrichment results. Histogram of the results of the KEGG enrichment analysis of the 594 differentially expressed genes (DEGs). The top 20 putative targets of pathway enrichment are shown.
