# Supplementary figures and images for: Antiviral Activity of Marine Bacterium Paraliobacillus zengyii Against Enterovirus 71 In Vitro and In Vivo
Source: Int J Mol Sci. 2025 Apr 8;26(8):3500. doi: 10.3390/ijms26083500 (PMC12026459; doi:10.3390/ijms26083500)

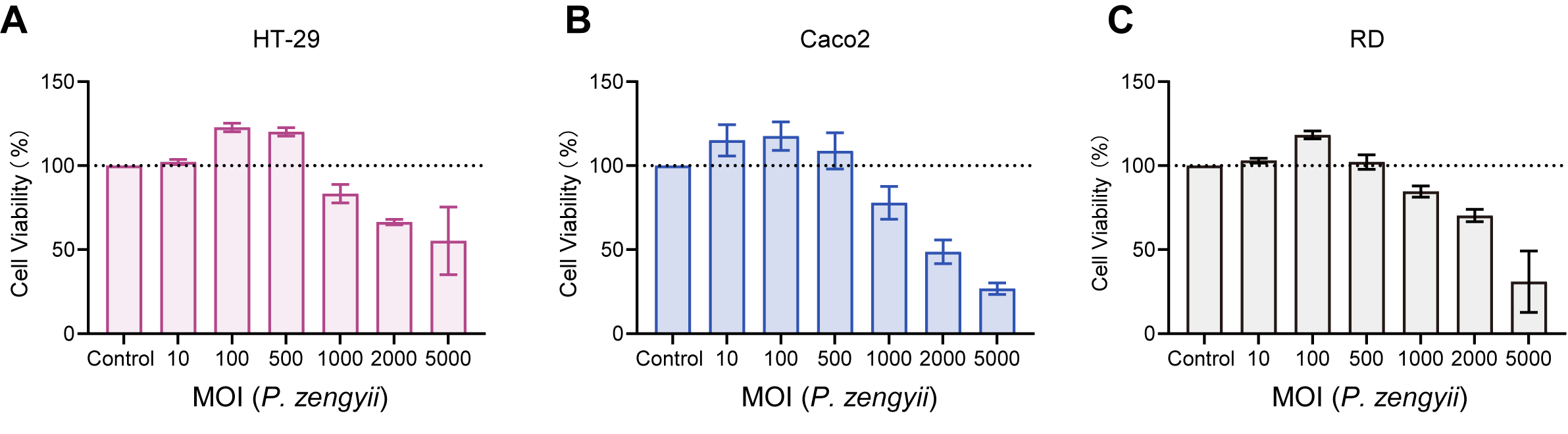

Supplement: Supplementary file 1 [file ijms-26-03500-s001.zip › Figure S1.tif]

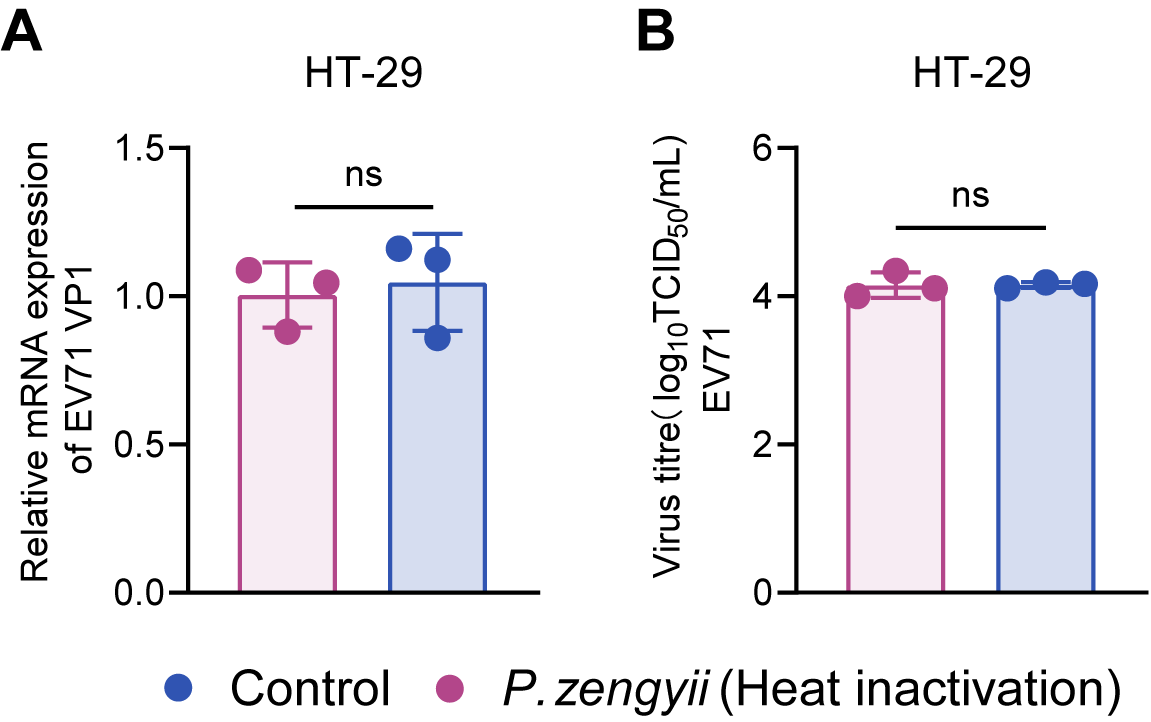

Supplement: Supplementary file 1 [file ijms-26-03500-s001.zip › Figure S2.tif]

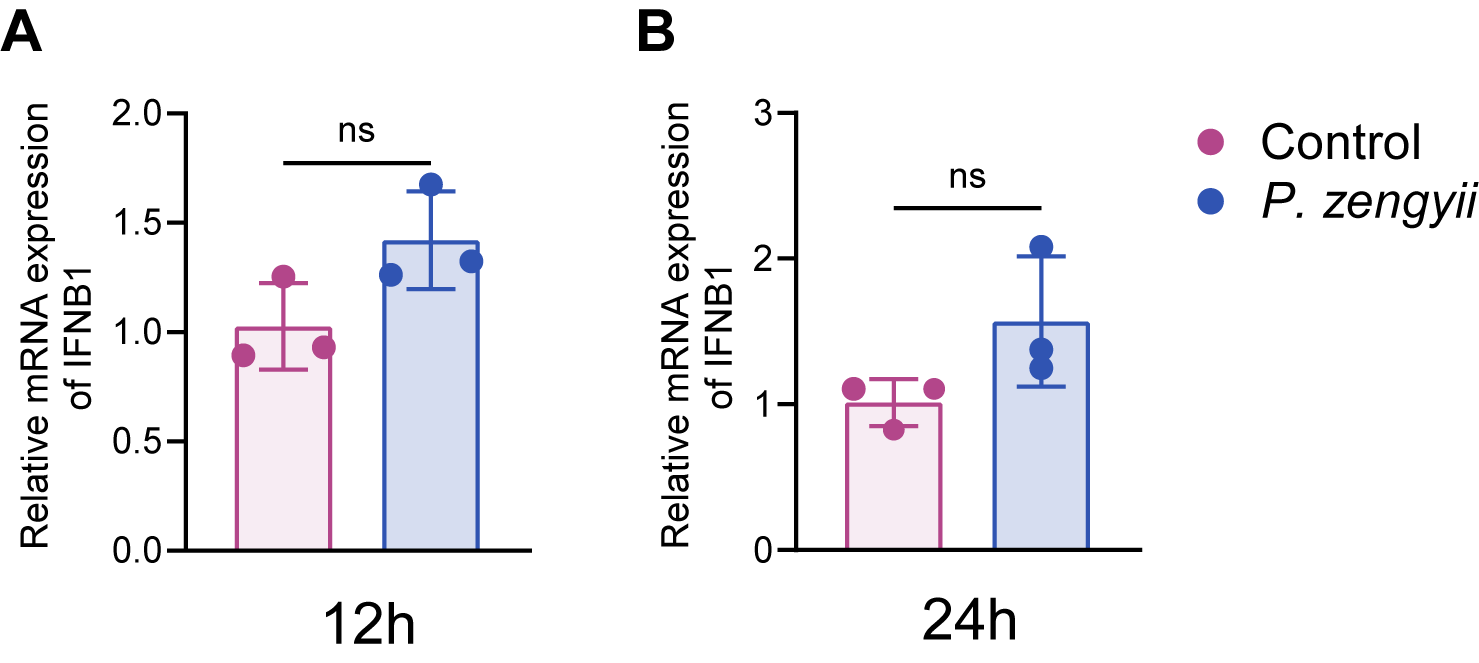

Supplement: Supplementary file 1 [file ijms-26-03500-s001.zip › Figure S3.tif]

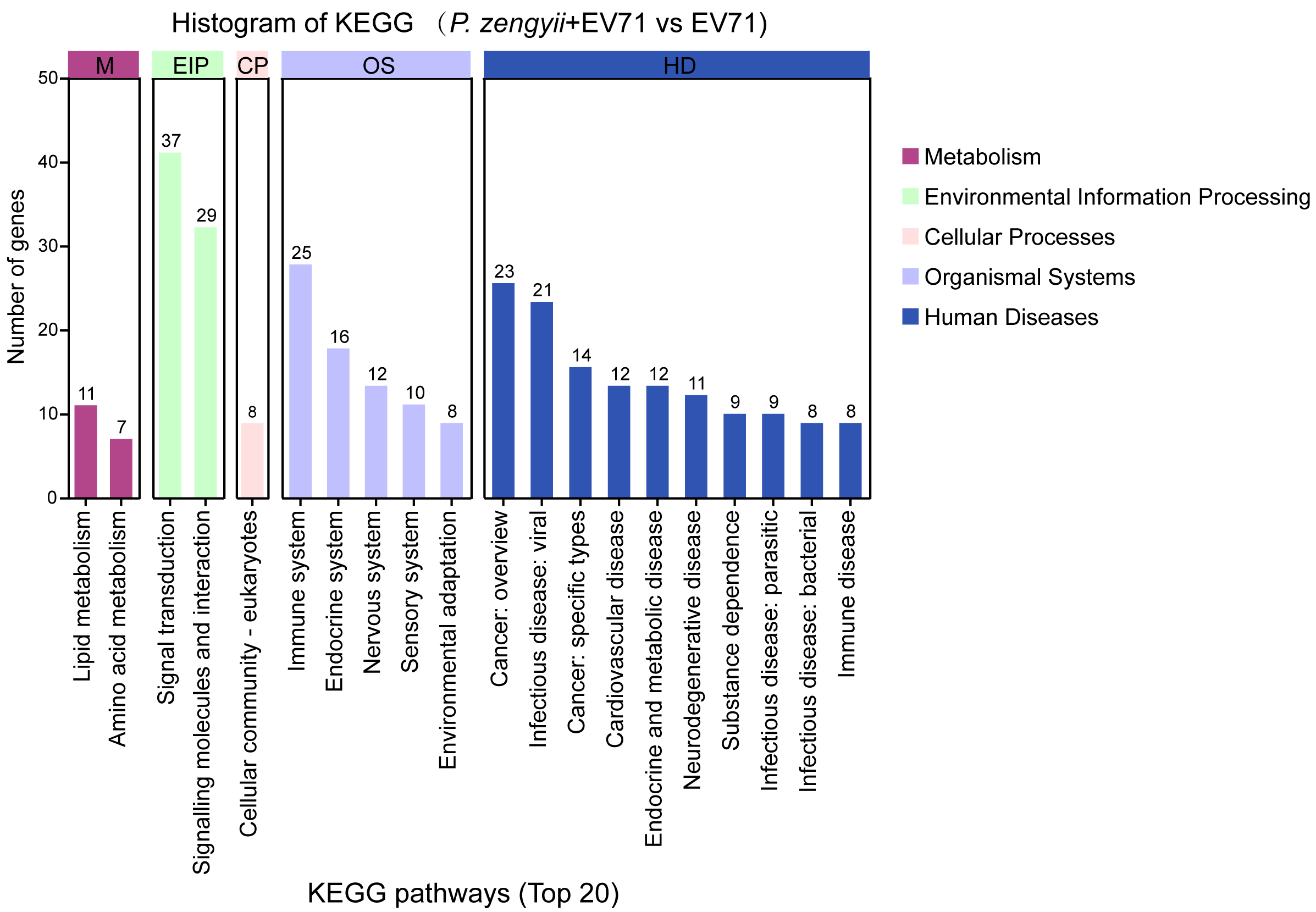

Supplement: Supplementary file 1 [file ijms-26-03500-s001.zip › Figure S4.tif]
